# Memories of emotional expressions in horses

**DOI:** 10.3758/s13420-018-0363-9

**Published:** 2018-10-18

**Authors:** Federica Amici

**Affiliations:** 1 Institute of Biology, Faculty of Life Science, University of Leipzig, Talstraße 33, 04103 Leipzig, Germany; 2 Junior Research Group of Primate Kin Selection, Department of Primatology, Max-Planck Institute for Evolutionary Anthropology, Deutscher Platz 6, 04103 Leipzig, Germany

**Keywords:** Cognitive ethology, Comparative cognition

## Abstract

Proops, Grounds, Smith, and McComb (2018) suggest that horses remember previous emotional expressions of specific humans, and use these memories to adjust their behavior in future social interactions. Despite some methodological shortcomings, this study raises important questions on the complexity of social interactions in nonhuman animals, which surely deserve further attention.

In their recent paper, Proops, Grounds, Smith, and McComb (2018) exposed horses (*Equus caballus*) to pictures of happy and angry facial expressions of specific humans. Horses responded more negatively when interacting with a person who previously had an angry facial expression, as compared with a person who had a happy expression. Therefore, like humans, horses appear to be able to form lasting memories of specific people’s emotional expressions, and use them in future social interactions to accordingly adjust their behavior.

Overall, this paper constitutes a first important step to understand whether animals remember previous facial expressions of specific individuals and, most crucially, whether they later use this information to efficiently navigate in their social systems. However, some methodological improvements might have helped the authors make a stronger claim. In their study, for instance, authors substantially reduced the generalizability of their findings by using emotional expressions of only two human models. Moreover, the authors increased the saliency of the emotional expressions shown to the horses by making the pictures larger than real models. However, this might have also facilitated horses’ detection of emotional cues, as images where larger and emotional cues easier to be seen. In this respect, the results of this study may tell us more about horses’ *potential* ability to read human emotional cues, rather than about their *actual* use of human facial expressions in everyday interactions, when horses may rely much less on visual cues (or may rather rely on cues other than visual ones) to assess human emotional expressions and accordingly adjust their behavior. Similarly, it is rather problematic that horses were not maintained in the same conditions during the time between exposure to the human picture and test phase: Different time intervals elapsed between exposure and test, and horses were returned to either stables or fields, so that very different conditions were likely experienced by individual horses, with possible unpredictable effects on their subsequent performance in the test phase.

Finally, and perhaps more crucially, the study presents no statistical tests directly comparing performance in the test and control conditions (or if these tests are presented, they must be nonsignificant; see Fig. 2 in Proops et al., 2018). In their study, the authors provide compelling evidence that some of the tested behaviors (e.g., time spent viewing the stimuli with a left or right gaze bias, time spent engaging in displacement behaviors) differ between individuals having observed angry and happy emotional expressions in the experimental condition (i.e., when the neutral human observed in the test phase was the one, whose emotional expression had been observed in the exposure phase). In contrast, the tested behaviors did not differ in the control condition (i.e., when the neutral human observed in the test phase was not the one, whose emotional expression had been observed in the exposure phase). However, these results are only suggestive of lasting emotional recognition in horses, and a much stronger case could be done by directly contrasting performance between experimental and control conditions, separately for angry and happy emotional expressions, as only control conditions constitute a sort of baseline against which experimental conditions should be tested for. In particular, only these statistical comparisons can control whether horses’ behavior was not simply a general response to the emotional expression shown in the exposure phase (i.e., angry or happy), but really a specific response to the particular human’s emotional expression observed during the exposure phase. From the data in their Fig. 2, it seems unlikely that such a direct statistical comparison between experimental and control conditions would confirm the results presented in the paper, at least for most behaviors.

That said, the study surely paves the way to more research in the field, and opens new exciting developments in the area of emotion recognition in animals other than humans. The authors, for instance, wisely decided to test horses’ ability to remember *human* emotional expressions: Given the long tradition of studies on emotional expressions in humans, this ensures methodological rigor when categorizing human expressions, in contrast to what happens with most nonhuman species. Moreover, given that horses are a domesticated species, this choice does not lack ecological validity. In the future, however, it would be especially interesting to test whether horses, and other animals, recognize emotional expressions of *conspecifics,* and keep track of them through time to decide their future social interactions. In this respect, Proops and colleagues (2018) are right, in stating that remembering emotional experiences with particular individuals may be adaptive for social bonding and aggression avoidance. Furthermore, the authors propose that keeping track of all these emotional experiences may require a combination of cognitive skills, ranging from individual identification, sensitivity to facial expressions, and memory for specific emotional experiences. In social groups with tens of individuals, or complex social dynamics (e.g., Aureli et al., 2008), however, this may easily overload the cognitive abilities of the individuals. Future studies should therefore assess whether these mechanisms are really cognitive, as suggested by the authors, and to what extent they may instead be subserved by more simple mechanisms, like emotionally based bookkeeping (see, e.g., Schino & Aureli, 2010).

Another interesting point is whether the ability to form lasting memories of specific individuals’ emotional expressions is limited to angry and happy expressions, or it extends to other ones. Depending on the species, for instance, it may also be adaptive to recognize fear, in order to avoid potentially dangerous situations. Furthermore, Proops and colleagues (2018) showed that memories of specific people’s emotional expressions are maintained up to 6 hours after exposure. In the future, it would be exciting to assess whether these memories are stored for longer time, and how sensitive they are to contrasting ones. For example, would the memory of an angry facial expression be stored even after days (something that may be useful, e.g., in species with very loose fission–fusion socialities, like orangutans), and how easily would it be “compensated” by subsequent exposure to a happy expression of the same individual? And how important are these emotional memories, as compared with, for instance, specific social events (e.g., aggressions, coalitions) directly involving subjects, or only third parties? The fact that horses’ heart rate differs while observing happy versus angry human faces (Smith, Proops, Grounds, Wathan, & McComb, 2016), but not when observing the same neutral human few hours after exposure (Proops et al., 2018), may suggest that, at least physiologically, the effect of emotional expressions on horses’ behavior is rather limited in time.

In brief, much more research needs to be done, in my opinion, before we can draw any conclusion on animals’ ability to remember and use emotional expressions of specific individuals in future social interactions. In a certain way, this study raises more questions than answers. If future researchers will rise to the challenge and try to address them, exciting new findings will likely await them.
